# Ligand Composition and Coating Density Co-Modulate the Chondrocyte Function on Poly(glycerol-dodecanedioate)

**DOI:** 10.3390/jfb14090468

**Published:** 2023-09-11

**Authors:** Yue Qin, Rhima M. Coleman

**Affiliations:** 1 Department of Biomedical Engineering, University of Michigan, Ann Arbor, MI 48109, USA; qiny@umich.edu; 2 Department of Mechanical Engineering, University of Michigan, Ann Arbor, MI 48109, USA

**Keywords:** ECM ligand coating, chondrocyte redifferentiation, poly(glycerol-dodecanedioate), cartilage tissue engineering, surface modification, scaffold design parameters

## Abstract

Inducing chondrocyte redifferentiation and promoting cartilaginous matrix accumulation are key challenges in the application of biomaterials in articular cartilage repair. Poly(glycerol-dodecanedioate) (PGD) is a viable candidate for scaffold design in cartilage tissue engineering (CTE). However, the surface properties of PGD are not ideal for cell attachment and growth due to its relative hydrophobicity compared with natural extracellular matrix (ECM). In this study, PGD was coated with various masses of collagen type I or hyaluronic acid, individually or in combination, to generate a cell–material interface with biological cues. The effects of ligand composition and density on the PGD surface properties and shape, metabolic activity, cell phenotype, and ECM production of human articular chondrocytes (hACs) were evaluated. Introducing ECM ligands on PGD significantly improved its hydrophilicity and promoted the chondrocyte’s anabolic activity. The morphology and anabolic activity of hACs on PGD were co-modulated by ligand composition and density, suggesting a combinatorial effect of both coating parameters on chondrocyte function during monolayer culture. Hyaluronic acid and its combination with collagen maintained a round cell shape and redifferentiated phenotype. This study demonstrated the complex mechanism of ligand-guided interactions between cell and biomaterial substrate and the potential of PGD as a scaffold material in the field of CTE.

## 1. Introduction

Articular cartilage is a specialized connective tissue present in synovial joints that provides a smooth surface for daily joint movement and load transmission. It is composed of highly specialized cells, chondrocytes, enclosed in the dense hyaline cartilage of their secretion. The hyaline cartilage enriches in collagen type II and proteoglycans, which contributes to the tissue function. Articular cartilage is of great clinical importance because its injury commonly leads to the development of osteoarthritis and can ultimately cause a significant musculoskeletal dysfunction; furthermore, it has a very limited self-regenerative or self-repair capacity due to a lack of nerves and blood vessels [1].

Autologous chondrocyte implantation (ACI) is a widely used treatment for osteochondral defects in the knee [2]. This technique involves harvesting chondrocytes from the non-weight-bearing zone of a patient’s joint and expanding them in a monolayer until a sufficient population of the chondrocytes is yielded for re-implantation into the damaged region. However, chondrocytes undergo dedifferentiation when cultured for a long period on tissue culture plastic (TCP) or some synthetic polymeric scaffolds, especially when cultured in low seeding density [3,4,5], leading to significant changes in their morphology and phenotype [6,7,8]. Losing the chondrocyte-differentiated phenotype impairs chondrocytes’ ability to regenerate robust cartilage in the defect of the knee, thus causing failure of the clinical treatment [2,9]. Therefore, chondrocyte-based therapies for cartilage defect repair aiming to guide chondrocyte redifferentiation has evoked intense interest in the field of cartilage tissue engineering [10].

Cell–matrix interactions play an important role in the maintenance of the differentiated chondrocyte phenotype. To mitigate chondrocyte dedifferentiation in monolayer culture, cartilage-specific ECM ligands are incorporated with *in vitro* culture strategies. Integrin attachment of the chondrocyte cytoskeleton to the ECM ligand has been implicated in the activity of various intracellular pathways, thus affecting chondrocyte genotype and phenotype [11,12,13]. As one of the dominant components of cartilage ECM, collagen interacts with chondrocyte via various integrin receptors. Previous studies have shown that coating collagen type I (Col I) on scaffold surfaces suppresses morphological changes in chondrocyte and improves ECM secretion in both a two-dimensional (2D) culture and a three-dimensional (3D) culture [4,14,15]. Another key articular cartilage-specific ECM ligand, hyaluronic acid (HyA), regulates chondrocyte function through interaction with many cell surface receptors. Previous studies have reported that HyA coating has a positive influence on chondrocyte function via a large number of cellular pathways including cell adhesion, phenotypic regulation, and cartilaginous matrix production [16,17,18]. When combined with Col I in hydrogels, HyA improved the beneficial effect of the ligand by stabilizing the chondrocyte phenotype and increasing proteoglycan synthesis [19]. Therefore, establishing a coating of HyA and/or Col I can be essential to incorporate more physiologically relevant biological cues into a culture environment for promoting cartilage regeneration. Moreover, different ligand densities may provide different levels of intracellular signals, thus differentially influencing cell function. Anecdotally, it is believed that high ligand density or concentration helps to maintain the round morphology of cells [20], which is widely accepted as a symbol of the matrix-anabolic phenotype of chondrocytes [6,21,22]. This means that, in addition to ligand composition, ligand density is another important parameter to consider for cartilage regeneration strategies.

Synthetic polyester elastomers have played a key role in cartilage tissue engineering [23]. To substitute the ECM in natural cartilage, these scaffolds are engineered to support chondrocyte attachment, proliferation, and redifferentiation and to guide neo-cartilage formation. Poly(glycerol-dodecanedioate) (PGD) is a novel biodegradable polyester elastomer, which acts as an attractive candidate for the scaffold materials in cartilage tissue engineering due to its biocompatibility, shape memory behavior, and non-linear elasticity that is similar to cartilage [24,25,26]. Additionally, the mechanical properties, geometry shapes, and degradation rates of the PGD scaffolds can be easily adjusted to match the cartilage tissue requirements [25,26,27]. The overall function of a scaffold depends on many scaffold parameters, including material characters, surface properties, scaffold topography, matrix stiffness, degradation rate, and so on [28,29,30,31]. Among those, one of the most crucial parameters is the surface interface properties, which directly regulate the micro-environmental cues presented by the scaffold and thus guide chondrocyte function [32]. However, the surface of polyester elastomer is sometimes not ideal for cell attachment and growth due to the material’s hydrophobicity and lack of cell growth factors on the surface [33,34]. Scaffolds composed of the above-mentioned ECM ligands create an environment that preserves the normal phenotype of cells to promote the regeneration of a cartilage-like matrix, unlike traditional 2D cultures, but the mechanisms behind this phenomenon are complex [35,36,37,38,39]. Therefore, introducing chemical and/or biological cues onto the surface of scaffolds, as well as improving their surface hydrophilicity, is one of the important tasks in engineering polyester scaffolds.

**Figure 1:**
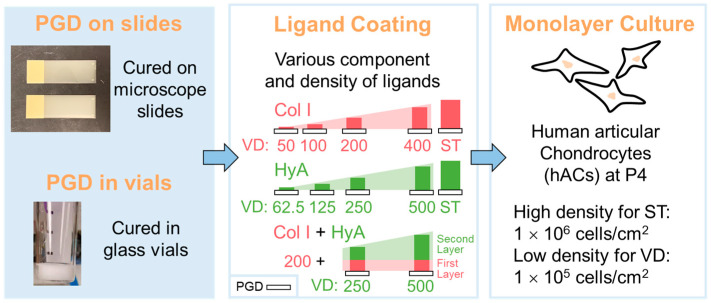
Schematic illustration of the experimental design. PGD was coated by different protocols of ligands, seeded with different densities of hACs, and then evaluated for surface properties and biological function. Col I: coating of collagen type I. HyA: coating of hyaluronic acid. ST: PGD with saturated coating. VD: PGD with various densities of coating, ranging from 200 mg to 500 mg (also indicated in Table 1).

Herein, this work focuses on modulating the surface parameters, i.e., ligand coating composition and density, to investigate their combinatorial effects on chondrocyte function. To develop an ECM-ligand-presenting environment for generating a biomaterial surface with enhanced cell affinity and ECM-anabolic ability, a novel biodegradable elastomer, PGD, was coated with varying densities of Col I and/or HyA (Figure 1). The surface morphology and hydrophilicity of the ligand-coated PGD were evaluated. Human articular chondrocytes (hACs) were cultured in a high-density monolayer on PGD with different types of ligand coatings for up to 4 weeks and examined at various time points to assess metabolic activity, cell morphology, and extracellular matrix production compared with TCP. Simultaneously, hACs were cultured in a low-density monolayer on PGD with different densities of ligand coatings and TCP, and cell morphology, cytoskeleton and focal adhesion, chondrocyte phenotype, and extracellular matrix production were assessed.

## 2. Materials and Methods

### 2.1. PGD Fabrication

PGD prepolymer was synthesized following the method described by Solorio et al. [27]. Briefly, PGD prepolymer was synthesized by mixing glycerol and dodecanedioic acid with a 1:1 molar ratio in a 120 °C flask under nitrogen and stirring conditions for 24 h, followed by vacuum environment at 120 °C for 24 h. The viscous prepolymer was then cast onto InkJet Plus microscope slides (Fisherbrand, Pittsburgh, PA, USA) or into 3 mL glass vials (Supelco, Bellefonte, PA, USA). The PGD prepolymer spontaneously spread to a film on the microscope slides or created a flat surface on the bottom of glass vials, and was then cured at 130 °C for 48 h. A vacuum was pulled and maintained at 90 mTorrs for the duration of the curing process. All the PGD samples were soaked in the growth media for 2 days with three media changes to remove any possible cytotoxic PGD byproducts that could dissolve in the media.

### 2.2. PGD Surface Coating

PGD surfaces were sterilized with sterile 70% ethanol through a 30 min ultrasonic wash before ligand coating. To analyze the effect of ligand composition on chondrocyte function, the ECM ligands collagen type I (Col I) or hyaluronic acid (HyA) were immobilized to PGD surfaces. Briefly, HyA powder (molecular weight ~1.5 mDa; Lifecore, Chaska, MN, USA) was dissolved in a sterile phosphate-buffered saline (PBS) to achieve a stock concentration of 0.25% (*w*/*v*). Col I from rat tail tendon dissolved in 0.1 M acetic acid solution (Corning, Bedford, MA, USA) was purchased for a stock concentration of 0.1% (*w*/*v*). The aqueous solution of HyA or Col I was pipetted onto the PGD film in a 4-well slide dish (Nunc rectangular dish, Thermo Scientific, Rochester, NY, USA) or PGD top surface in a 3 mL glass vial until the PGD surface was fully immersed to reach saturation. All samples were completely air-dried for 2 days in a sterile environment at room temperature to prepare the substrates with saturated coating (ST).

To analyze the effect of ligand density on chondrocyte function, a serial desired concentration of Col I or HyA on PGD was made from diluting sterile filtered stock solution. The aqueous solution of HyA or Col I was pipetted onto PGD film in a 4-well slide dish or PGD top surface in a 3 mL glass vial in calculated volume to reach the designed mass (Table 1). All samples were completely air-dried for 3 days at room temperature to prepare the substrates with various densities of coating (VD). A layer-by-layer strategy was utilized to coat the combination of Col I (first layer) and HyA (second layer) onto PGD to improve HyA retention levels before seeding the cells.

### 2.3. Surface Characterization

#### 2.3.1. Ligand Retention Level

PGD on slides or in vials with various densities of coating was immersed in 1 mL phosphate-buffered saline (PBS) at 37 °C for 24 h, and then the supernatant solution was collected to run gel permeation chromatography (GPC). Standard Col I and HyA solutions varying in concentration were prepared in PBS. Gel permeation chromatography was carried out using a refractive index detector. Both supernatant samples and standard solutions were run through the column at a flow rate of 0.6 mL/min. Col I eluted around the 9–10 min mark, while HyA eluted around the 7–12 min mark, and the total run lasted 20 min. The peak area associated with each eluting peak was quantified to calculate the concentration of Col I or HyA in the supernatant solution, represented by $C_{\mathrm{elute}}$. The ligand retention level was calculated by the following equation, where $C_{\mathrm{initial}}$ represented the initial coating concentration corresponding to the designed mass in Table 1. The ligand retention level is also known as the mean coating efficiency of each ligand, representing the percentage of ligand remaining on the PGD surface after PBS immersion.

Ligand retention level = Coating efficiency = (*C_initial_* − *C_elute_*)/*C_initial_* × 100% (1)

#### 2.3.2. Surface Topography

The surface morphology of coated PGD samples was characterized by scanning electron microscopy. PGD samples were sputtered with a 10 nm thick layer of gold (Leica EM ACE200, Wetzlar, Germany) prior to being observed on a field emission scanning electron microscopy (JEOL JSM 7800F, Tokyo, Japan).

#### 2.3.3. Water Contact Angle

The surface wettability of uncoated PGD, coated PGD, and TCP was comparatively evaluated by a water contact angle measurement using a sessile drop method [40]. The apparent water contact angle of the samples was tested by a DSA100 instrument (Krüss GmbH, Hamburg, Germany). The water contact angle was measured immediately after depositing 5 μL of an ultrapure water droplet on the substrate surface by applying a circular fit of the drop and determining the tangent at the intersection.

### 2.4. Cell Seeding and Culture Conditions

Human articular chondrocytes (hACs) from a healthy young male donor (age 19, CELLvo, StemBioSys, https://www.stembiosys.com/, accessed on 10 June 2019) were expanded in flasks with growth medium: Low glucose Dulbecco’s Modified Eagles medium (Gibco, Grand Island, NY, USA) supplemented with 10% fetal bovine serum (FBS), 1% antibiotic-antimycotic, 1 ng/mL transforming growth factor beta-1 (TGF-β1), 10 ng/mL platelet-derived growth factor-BB (PDGF-BB), and 5 ng/mL fibroblast growth factor-2 (FGF-2). The hACs were subcultured to passage 4 (P4) using growth medium at 37 °C, 5% CO_2_, while at each passage the cells were expanded until the plate reached confluency. The hACs (at P4) were seeded onto the top surface of coated PGD, PGD without coating, and TCP in the 4-well slide dishes or 3 mL glass vials and then incubated at 37 °C for 3 h for cell subsidence (Figure 1). High seeding density of 1 × 10^6^ cells/cm^2^ was applied in saturated coating (ST) groups, while low seeding density of 1 × 10^5^ cells/cm^2^ was applied in various density coating (VD) groups. The redifferentiation medium was then added to replace the growth medium, which was formulated as high glucose Dulbecco’s Modified Eagles medium (Gibco, Grand Island, NY, USA), 1.25 mg/mL bovine serum albumin (BSA), 1% antibiotic-antimycotic, 1% ITS+Premix (Corning, Bedford, MA, USA), 10 ng/mL TGF-β1, 40 µg/mL L-proline, 50 μg/mL ascorbic acid 2-phosphate, 1 mM sodium pyruvate, 100 nM dexamethasone, and 10 mM HEPES. The hAC-seeded PGD and TCP were then cultured in the humidified incubator with 5% CO_2_ at 37 °C for 2, 21, or 28 days.

### 2.5. Cell Attachment Analysis

To analyze the effect of ECM ligand composition on chondrocyte shape, hACs were seeded at a high density onto the following surfaces: PGD with saturated coating, PGD without coating, and TCP. These were cultured for 2 days as described above. The surface morphology of seeded PGD samples after 2 days of culture was characterized by scanning electron microscopy with the same method that is described above. Additionally, the samples were examined by F-actin staining using phalloidin TRITC (Sigma-Aldrich, St. Louis, MO, USA) following the manufacturer’s instruction. Briefly, the samples were fixed with 10% neutral buffered formalin for 30 min, were permeabilized by 0.5% Triton X-100 (Sigma-Aldrich, St. Louis, MO, USA) in Tris-buffered saline (TBS) for 5 min, and then were blocked with 10% goat serum and 1% BSA in TBS for 1 h at room temperature. The samples were strained with 300 nM phalloidin solution for 30 min and then were sealed by coverslip with one drop of anti-fade mounting medium. The images of hACs were captured on the fluorescence microscope and Nikon A1 Confocal microscope (Nikon, Tokyo, Japan). To assess cell attachment, the perimeter and area of each human articular chondrocyte from 5 images of each group were quantified by analysis of the images via ImageJ (National Institute of Health, Bethesda, MD, USA). The perimeter and cell area of each measured chondrocyte were collected by “Analyze > Measure” command after segmenting each cell [41]. The circularity of each cell is defined by the following equation:

Circularity = 4π × Area/Perimeter^2(2)

To assess the effect of ligand density on chondrocyte attachment, a low density of human articular chondrocytes (1 × 10^5^ cells/cm^2^) was seeded onto coated or uncoated PGD surfaces, and was then cultured for 2 days as described above. Samples were then examined by F-actin staining using phalloidin TRITC following the same processes as described above. Simultaneously, samples were marked by nucleus and focal adhesion protein staining using DAPI (Invitrogen, Carlsbad, CA, USA) and vinculin monoclonal antibody (VLN01, MA5-11690; Invitrogen, Carlsbad, CA, USA) separately, following the manufacturer’s instruction. Briefly, the PGD samples were incubated overnight at room temperature with 2 μg/mL mouse vinculin monoclonal IgG followed by 2 h incubation with 2 μg/mL Alexa Fluor 488 goat anti-mouse IgG (Invitrogen, Carlsbad, CA, USA). The samples were counterstained with 300 nM phalloidin solution for 30 min and then 300 nM DAPI for 5 min. Images of hACs were taken from Nikon A1 Confocal microscope.

### 2.6. Metabolic Activity Analysis

Chondrocyte metabolic activity was analyzed via Cell Counting Kit-8 (CCK-8, Dojindo, Kumamoto, Japan) following the manufacturer’s instruction. Briefly, hACs seeded on both uncoated and coated PGD (only ST groups) were cultured in glass vials in 28-day culture in differentiation media. Pure PGD without seeded cells was set as control group to decouple the effect of PGD degradation on CCK-8 reactivity during the entire culture. One hundred microliters of CCK-8 solution was added to each vial on days 2, 5, 8, 12, 15, 20, 25, and 28 and the vials were incubated at 37 °C for 4 h. The absorbance of the solution in each vial was measured at 450 nm.

### 2.7. Biochemical Analysis

To analyze the effect of ECM ligands coating on sulfated glycosaminoglycan (sGAG) production, 1,9-dimethyl methylene blue (DMMB) assay was conducted on TCP and all the hAC-seeded PGD groups (both ST and VD groups) cultured in glass vials after 28-day culture as previously described [42,43]. Briefly, the matrix generated on the top surface of PGD or TCP during the culture was digested with papain [42]. The absorbance of the samples after the addition of DMMB dye was measured at 525 nm and 595 nm and compared with chondroitin sulfate standards [43].

### 2.8. Chondrocyte Phenotype Analysis

To assess chondrocyte phenotype, after 21 days of culture, the PGD samples were immunofluorescently labeled for 1 h at room temperature either with 2 μg/mL mouse monoclonal IgG collagen type II (Invitrogen, Carlsbad, CA, USA) followed by 2 μg/mL Alexa Fluor 647 goat anti-mouse IgG (Invitrogen, Carlsbad, CA, USA), or with 2 μg/mL rabbit monoclonal aggrecan (Invitrogen, Carlsbad, CA, USA) followed by 2 μg/mL Alexa Fluor 488 donkey anti-rabbit IgG (Invitrogen, Carlsbad, CA, USA). Images of hACs were taken on a Nikon A1 Confocal microscope.

### 2.9. Histological Analysis

High seeding density model was used in VD groups to assess the matrix distribution in long-term monolayer culture on PGD. After 28 days of culture, the cell-seeded PGD was collected and fixed with 70% ethanol for 45 min followed by washing with 3% acetic acid solution for 5 min. The fixed samples were stained with 1% Alcian blue dye in 3% acetic acid (pH = 2.5, Poly Scientific, Bay Shore, NY, USA) for 16 h at 4 °C and then washed with water to observe aggrecan accumulation on PGD. All the stained samples were imaged using a light microscope.

### 2.10. Statistical Analysis

Unless otherwise indicated, results were analyzed using a one-way ANOVA and Tukey post hoc test for multiple comparisons in GraphPad Prism 8.0.1 (GraphPad Software, San Diego, CA, USA), and data were represented as mean ± standard deviation. The water contact angle, cell area, circularity, metabolic activity, and sGAG production of hAC cultured on PGD were compared across different coating groups, uncoated PGD, and TCP control. The criterion for statistical significance was *p* < 0.05 in all tests. Sample sizes (*n*) are indicated in the corresponding figure legends.

## 3. Results

### 3.1. Influence of Ligand Coating on Surface Properties of PGD

**Figure 2:**
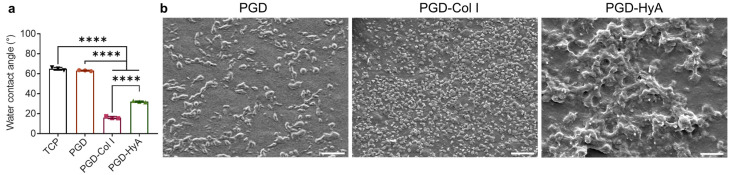
Ligand coating altered the surface properties of PGD. (**a**) Water contact angles of TCP, PGD without coating, and PGD coated with different types of saturated ligands (*n* = 3). Significant differences are indicated by **** *p* < 0.0001. PGD: pure PGD surface without coating. PGD-Col I: PGD coated with saturated collagen type I. PGD-HyA: PGD coated with saturated hyaluronic acid. (**b**) SEM images of the surface topography of PGD coated with each saturated ligand (3000×). Scale bar: 5 μm.

The water contact angles determined on the uncoated PGD and TCP were approximately 60° (Figure 2a). The surface wettability of the PGD surfaces was significantly enhanced after coating with saturated Col I and HyA, while the Col I-coated PGD was the most hydrophilic.

The surface morphology of uncoated and coated PGD was examined by scanning electron microscope (SEM). The ligand coatings altered the surface topography and roughness of the PGD (Figure 2b). PGD without coating had a slightly rough surface with the presentation of a large number of spikes with dimensions ranging from 1 to 5 μm. Coating with saturated collagen type I increased the number of spikes presented on the surface and decreased the overall size of the spikes (less than 1 μm). When coated with saturated hyaluronic acid, the PGD surface exhibited increased roughness with many micro-hillocks and micro-scale valleys that were larger in dimension (>5 μm), compared with the other two groups. Overall, Col I coating increased PGD surface roughness by generating large numbers of smaller-sized spikes, while HyA coating increased surface roughness by creating large hillocks.

### 3.2. Influence of Ligand Coating Composition on Chondrocyte Shape

**Figure 3:**
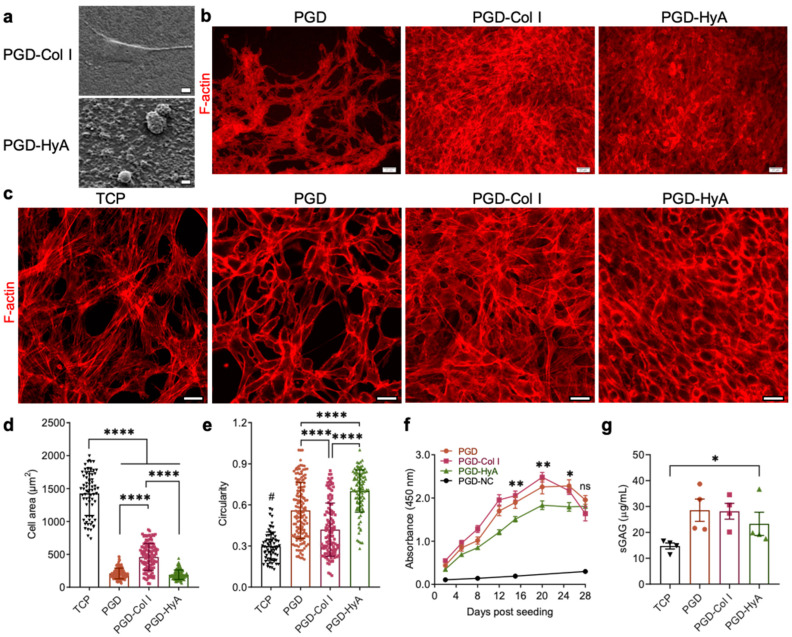
ECM ligand composition influenced chondrocyte behavior on PGD surfaces. (**a**) SEM images of the morphology of chondrocytes that were seeded on saturated ligand coating of PGD (1000×). Scale bar: 1 μm. (**b**) Fluorescence images of phalloidin TRITC staining (F-actin, red) on chondrocytes-seeded PGD films with no coating (PGD), saturated Col I coating (PGD-Col I), or saturated HyA coating (PGD-HyA) after 2 days of culture. Scale bar: 20 μm. (**c**) Confocal images of phalloidin TRITC staining (F-actin, red) on chondrocyte-seeded PGD films with different saturated coatings after 2 days of culture. TCP denotes tissue culture plastic. Scale bar: 20 μm. (**d**) Quantification of the area of each hAC on coated PGD (*n* = 70). Significant differences are indicated by **** *p* < 0.0001. (**e**) Quantification of the circularity of each hAC on coated PGD (*n* = 70). Significant differences among PGD, PGD-Col I, and PGD-HyA are indicated by **** *p* < 0.0001. Significant differences between TCP and other groups are indicated by # *p* < 0.0001. (**f**) Metabolic activities of hACs on PGD coated with different saturated ligands in 28-day culture (*n* = 8). PGD-NC denotes PGD with no cells seeded. Significant differences among PGD, PGD-Col I, and PGD-HyA on the same day of culture are indicated by * *p* < 0.05 and ** *p* < 0.01, ns: not significant. (**g**) The sGAG production of hACs on PGD coated with different saturated ligands after 28 days of culture (*n* = 4). Significant difference between PGD groups and TCP is indicated by * *p* < 0.05.

The hACs attached and proliferated on the PGD films, indicating good cytocompatibility (Figure 3a,b). According to SEM images of the cell-seeded PGD, a saturated Col I coating led to spread cell shape of hAC with a larger cell area, while saturated HyA coating resulted in a round cell shape and a smaller cell area on day 2.

F-actin-stained images further confirmed that the cell morphology was regulated by ligand compositions (Figure 3b,c). The cells on the Col I- and HyA-coated films were 100% confluent with dense, multi-layered structures of cells, while the uncoated PGD and TCP showed fewer cells with spaces between cell clusters, resulting in a honeycomb-like structure. This suggests that ligand coatings improved hACs proliferation or attachment. HyA-coated PGD film retained more hACs with a round cell shape compared with the Col I-coated group. The hACs grown on collagen type I-coated PGD exhibited a flat, stretched polygonal shape with high numbers of stress fibers, which was also found on TCP. In the HyA group, cells were smaller and rounder, and the actin fibers were distributed evenly beneath the cell membrane, which is associated with the maintenance of the chondrocyte differentiated phenotype. This was supported by quantification of cell area and circularity via ImageJ analysis (Figure 3d,e), which showed that Col I coating on PGD films increased hACs’ spreading, with the largest cell area and lowest circularity, while HyA coating maintained the smaller and rounder cell shape that are typically found in a 3D culture.

### 3.3. Influence of Ligand Coating Composition on Chondrocyte Anabolic Activity

The impact of ECM ligand composition on hACs’ metabolic activities (Figure 3f) and ECM production (Figure 3g) after 28-day culture was analyzed. The metabolic activity of hACs in all cell-seeded groups increased during the culture, indicating good cytocompatibility and support for cell growth. The metabolic activity of hACs varied depending on the PGD surface (Figure 3f). The metabolic activity of hACs cultured on Col I-coated PGD was the highest, while that of those cultured on HyA-coated PGD was the lowest; however, this changed after day 20. From day 20 to day 28, hACs cultured on PGD-Col I exhibited gradually decreased metabolic activity, whereas hACs cultured on PGD-HyA maintained their metabolic activity level. hACs cultured on uncoated PGD maintained a stable level of metabolic activity from day 20 to 24, which then decreased by day 28. There were no significant differences in the metabolic activity between the PGD, PGD-Col I, and PGD-HyA groups on day 28. PGD controls with no cells (PGD-NC) exhibited an absorbance much lower than other groups throughout the culture period, suggesting that PGD degradation byproducts had no effect on the accuracy of this assay. According to Figure 3g, all the PGD groups supported chondrocyte anabolic function. Chondrocytes cultured on PGD produced more sGAG on day 28 than those on TCP, while there was no significant difference in the sGAG production from chondrocytes among uncoated, Col-coated, and HyA-coated PGD.

### 3.4. Combinatorial Effect of Ligands Composition and Density on Chondrocyte Function

**Figure 4:**
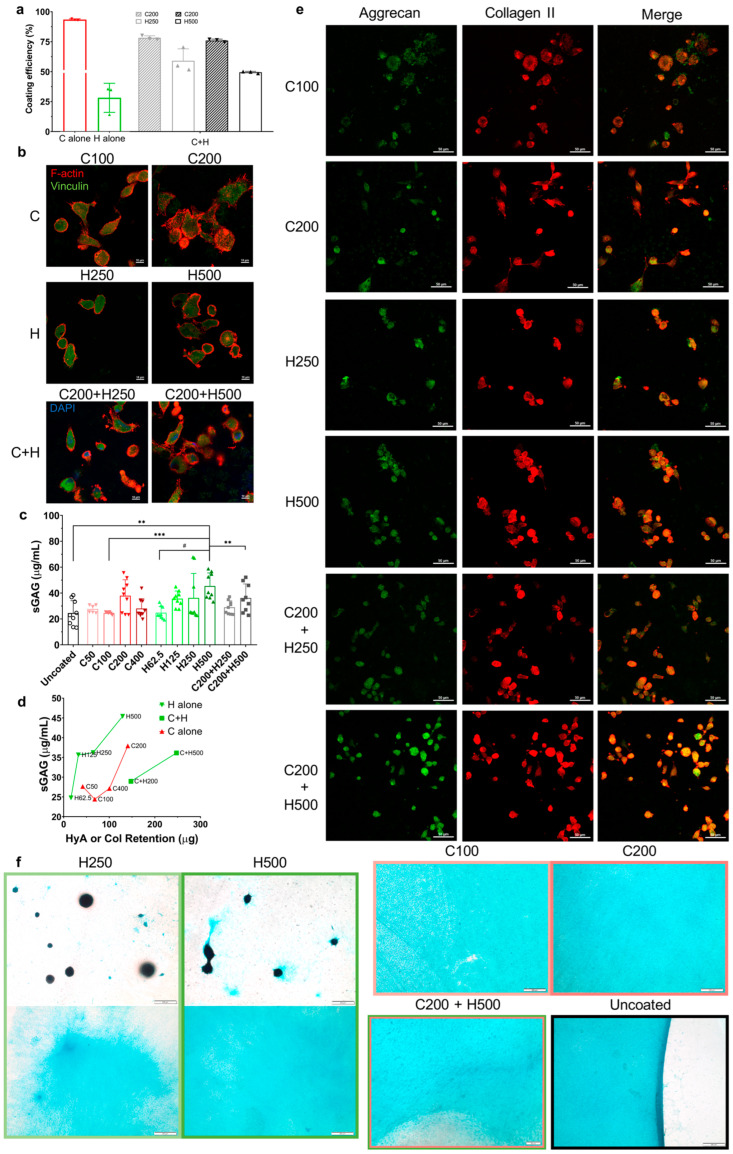
ECM ligands composition and density co-modulated chondrocyte behavior on PGD surfaces. (**a**) Ligand retention level of various ligand compositions after 24 h immersion in PBS (*n* = 3). C100 or C200: 100 μg or 200 μg Col I was coated onto PGD before immersion. H250 or H500: 250 μg or 500 μg HyA was coated onto PGD before immersion. (**b**) Fluorescence images of phalloidin TRITC staining (F-actin, red), DAPI staining (nucleus, blue), and vinculin staining (focal adhesion protein, green) on chondrocytes-seeded PGD with various ligand compositions and densities. Scale bar: 10 μm. (**c**) The sGAG deposition of hACs on PGD with various ligand compositions and densities in 28-day culture (*n* = 9). Significant difference is indicated by ** *p* < 0.01, *** *p* < 0.001, # *p* < 0.0001. Uncoated: PGD without ligand coating. (**d**) Correlations between ligand profile and ECM production (*n* = 9). The x-axis: ligand retention level. (**e**) Immunofluorescent images for collagen type II and aggrecan in hACs grown on coated PGD. Scale bar: 50 μm. (**f**) Alcian blue staining for the distribution of accumulated aggrecan after 28-day culture. Scale bar: 50 μm (C200+H500) or 200 μm.

According to the GPC analysis of coated PGD with various coating densities, approximately 90% Col I or 25% HyA remained on the PGD after 24 h of immersion in PBS (Figure 4a). The ligand retention level (coating efficiency) of each ligand is represented by the percentage of ligand remaining on the PGD surface when coated, dried, and then immersed in PBS for 24 h. Compared with HyA alone, combined Col I and HyA (C+H) using a layer-by-layer strategy doubled the HyA retention level to above 50%. Compared with Col I alone, C+H group slightly dampened Col I retention.

Immunofluorescent staining of F-actin and staining for focal adhesion protein was conducted to evaluate the influence of the ligand coating profile of PGD on the cell shape (Figure 4b). The hACs attached and proliferated on coated PGD films, indicating their cytocompatibility. The cell morphology was regulated by both ligand composition and density during 2 days of culture. The hACs grown on HyA-coated PGD and C+H-coated PGD had a rounder cell shape, representing a chondrogenic phenotype, than Col I-coated PGD. The hACs grown on PGD with C200 coating showed the most stretched polygonal shape with the largest cell area, suggesting potential changes in the phenotype of the chondrocytes. The hACs proliferated on PGD with combined Col I and HyA coating exhibited the smallest cell area. Increasing ligand density led to an increased single-cell area of chondrocytes and an increased number of cells in all the groups. A lower density of each ligand composition led to more spindle cell shapes instead of round or polygonal cell shapes compared with higher ligand density. Vinculin is a cytoskeletal protein associated with focal adhesion and adherent junctions, which functions in adhesion and/or signaling between the extracellular environment and the cell [44]. Vinculin staining was widely presented inside the contour line of F-actin staining in all the groups, suggesting focal adhesion units were highly expressed in hACs at the interface of the ligand-coated PGD.

Analysis of sGAG production highlighted the combinatorial effects of ligand composition and density on chondrocyte ECM production on PGD (Figure 4c,d). According to Figure 4b, among all the groups, H500 exhibited the highest sGAG accumulation. Moreover, a higher HyA initial coating density resulted in enhanced ECM production, supporting the claim that the ligand density of HyA was positively related to hAC anabolic activity in 28-day cultures. There were significant differences in sGAG production levels between the different initial densities of Col I, as chondrocyte’s ECM production on Col I-coated PGD was highest on surfaces initially coated with 200 μg. Combining collagen and hyaluronic acid by layer-by-layer coating method resulted in different effects on ECM production compared with the individual coatings (Figure 4c). With the same initial density of HyA, the C200+H500 group showed significantly lower sGAG production than the H500 group. However, the C200+H250 group showed no significant difference in sGAG production compared with the H250 group. Overall, we found that a high density of hyaluronic acid coating significantly increased sGAG synthesis, and the addition of Col I coating significantly reduced sGAG production.

Additionally, the correlation between the ligand retention level and ECM production was determined as shown in Figure 4d. There was a dose-dependent response of sGAG accumulation to the retention of hyaluronic acid or collagen type I alone for hACs grown on coated PGD. Generally, the sGAG production was positively related to the retention of HyA or Col I alone. HyA-coated PGD had a higher sGAG production than Col I-coated and Col I + HyA-coated PGD, regardless of ligand coating density. Moreover, with similar ligand retention levels (H500, C200, and C200+H250), HyA alone had a larger effect on improving sGAG production compared with other compositions, suggesting that a higher binding efficiency does not necessarily induce higher ECM production, which supported the combinatorial effect of both ligand composition and density on chondrocyte’s ECM production on PGD.

The chondrocyte phenotype was analyzed by immunostaining for collagen type II and aggrecan after 21 days of monolayer culture (Figure 4e). Collagen type II and aggrecan were co-localized in the peri-nuclear cytoplasm of hAC. Chondrocytes grown on all the ligand-coated groups showed both collagen type II and aggrecan immunostaining, suggesting these chondrocytes maintained or regained their differentiated phenotype with anabolic activity for the dominant structural macromolecules of articular cartilage. Higher ligand density exhibited a higher number of cells that secreted both collagen type II and aggrecan, while the C200+H500 group exhibited the largest numbers of cells co-localized with both macromolecules among all the groups.

The distribution of deposited aggrecan from hACs during 28 days of monolayer culture on VD-coated PGD was regulated by the ligand composition and HyA density, which was illustrated in the Alcian blue staining results (Figure 4f). Hyaluronic acid coating generated two distinct neo-matrix distributions. The chondrocytes grown on HyA coating of PGD either formed the scattered 3D cell pellets with dense staining or a homogeneous 2D membrane of aggrecan, while the ligand density only impacted the area and thickness of the aggrecan membrane instead of the shape and size of the pellets. On the contrary, various densities of collagen consistently directed the hACs to secrete the homogeneous distribution of aggrecan film on PGD. It should be noted that introducing collagen type I prior to hyaluronic acid coating led to a 2D aggrecan deposition and vanished the cell pellets. The uncoated PGD created a denser and more homogeneous distribution of aggrecan with clearer boundaries than the coated groups did.

## 4. Discussion

Prolonged monolayer expansion of chondrocytes is a necessary process in the clinical repair of large cartilage defects using an autologous chondrocyte implantation procedure. The phenotype of chondrocytes may change dramatically when cultured for a long period on a tissue culture plate, which is known as chondrocyte dedifferentiation. In this circumstance, the gene expression of chondrocytes shifts from a differentiated phenotype to another resembling that of fibroblasts, which causes decreased secretion of crucial structural components of articular cartilage (collagen type II and aggrecan) and increased production of type I collagen, fibronectin, and small non-cartilaginous proteoglycans [7]. It also results in a change in the actin cytoskeletal configuration, and thus, alters the cell shape: chondrocyte shifts from a round towards a fibroblastic shape [9]. In the end, the quality of the matrix synthesized by the chondrocytes deteriorates significantly from that of normal cartilage. In this work, we enhanced the ability of hACs to synthesize a cartilaginous ECM and promoted hAC redifferentiation on PGD during monolayer culture by developing a cell-favorable surface environment using ligand coating.

To overcome the chondrocyte phenotype switch during monolayer culture, cartilage tissue engineering has explored several ways to prevent chondrocyte dedifferentiation or promote chondrocyte redifferentiation, such as integrating biological cues on the substrates for a monolayer culture, plating chondrocyte in a high-density monolayer culture, and seeding cells in a 3D scaffold/hydrogel. While various hydrogels consisting of cartilage ECM components do support the maintenance of the chondrocyte phenotype, they cannot fulfill the protective mechanical function of the articular cartilage [45]. Biodegradable polyesters are usually mechanically tough, but their hydrophobicity leads to inefficient cell attachment, requiring surface modification to enhance their cell affinity [46]. PGD is a novel biomaterial whose potential as a scaffold for cartilage tissue engineering has never been studied. Our findings demonstrated that PGD, alone or in combination with ECM ligands, promoted chondrocyte survival, attachment, and ECM production (Figure 3 and Figure 4). The large pores of the porous polyester scaffold usually provide a 2D environment for cell attachment due to a much larger magnitude of pore size compared with a single cell size, and the 2D culture on stiff substrates (like TCP) may lead to aberrant chondrocyte phenotypes and loss of the differentiated phenotype. Additionally, the intrinsic hydrophobicity and lack of biological adhesion sites are the shortcomings of polyester biomaterials, e.g., PGD. Incorporating synthetic biomaterials with ECM ligands has been used to enhance the surface hydrophilicity and cell affinity, which play an important role in cell adhesion and growth on scaffolds [47]. Ligand coatings significantly increased the surface wettability of PGD (Figure 2) due to the richness of polar functional groups in hyaluronic acid [48] or the hydrocarbon chains and hydrophilic functional groups in collagen molecules [49]. Moreover, coating specific cartilage ECM ligands onto PGD facilitated the formation of a round cell shape and the production of sGAG, however, this beneficial effect is dependent on ligand composition (Figure 3 and Figure 4).

This study commenced with exploring the effect of the ECM ligand composition on chondrocyte function by functionalizing PGD with saturated ligand coating. Overall, our results revealed that incorporating ECM ligands with PGD can help maintain round chondrocyte morphology and its differentiated phenotype, which could be supported by the cell–ligand binding complex. The binding of surface receptors to ligands is the molecular basis of the initial adhesion of transplanted chondrocytes to surrounding cartilage in the defect site [50]. After initial attachment, the integrin on the chondrocytes is involved in proliferation, survival, differentiation, matrix remodeling, and response to mechanical stimuli [35]. To be specific, chondrocytes express integrin receptors, for example, α1β1 and α10, which fulfill a function in binding to collagen ligands. Studies have shown that even with a high population-doubling number, chondrocytes on the pure collagen type I substrate were round-shaped and produced collagen type II, whereas the ones on the TCP substrate chondrocytes lost their differentiated phenotype [4]. However, our results showed a high seeding density of hACs cultured on collagen type I-coated PGD still grew into a flattened, stretched shape that resembled those on TCP (Figure 3c). This was possibly caused by the higher stiffness of PGD compared with Col I substrate, since it has been shown that stiff matrices induce the increased cell traction forces applied to the substrate and a large flattened morphology of chondrocytes [27,51]. Conversely, our results showed that hyaluronic acid coating of PGD successfully induced a round hAC morphology (Figure 3c) and higher synthesis of sGAGs compared with uncoated and other ligand-coated surfaces (Figure 4c). Previous studies have shown that the actin cytoskeleton is a major determinant of chondrocyte shape, and the standard actin features of cells such as stress fibrils, filipodia, and lamellipodia are features of dedifferentiated chondrocytes cultured in monolayer [13,52]. Hyaluronic acid-coated PGD retained the rounded morphology of the hACs without showing these actin features on day 2 and maintained high metabolic activity and anabolic activity during 28 days of culture, demonstrating the potential of the microenvironment provided by HyA-coated PGD to preserve chondrocyte phenotype. This may be explained by previous studies, which showed that cell binding to hyaluronic acid, via surface receptors including CD44 and RHAMM, triggers a sophisticated signaling pathway causing chondrocytes to maintain their natural phenotype [38]. Different ligand coating compositions resulting in different chondrocyte behavior could also be driven by the alteration of surface morphology (Figure 2b), since research has shown that human chondrocytes maintained their phenotype on the surface with a large diameter of hillocks (>5.67 μm, similar to our HyA-saturated coating) [53]. Additionally, compared with Col I, HyA coating guided the distribution of matrix deposition for hACs differently during the 28-day culture by mediating unstable adhesion of cells on the substrate due to a lack of integrin binding [54], thus generating more 3D cell pellets with dense Alcian blue staining instead a of 2D matrix (Figure 4f), which could assist in chondrocyte redifferentiation in 3D scaffold cultures and long-term *in vitro* cultures. Through incorporating adhesive ligands such as Col I into the HyA, chondrocyte gained the ability to form attachments and produced aggrecan membrane on PGD. Therefore, these results suggested that applying hyaluronic acid to the coating of polymeric substrate, e.g., PGD, is a good way to improve the chondrogenic ability of scaffolds in cartilage tissue engineering. Furthermore, despite its hydrophobicity, the uncoated PGD surface maintained a rounder cell shape and induced higher ECM production compared with TCP (Figure 3c,g), indicating its potential for improving cartilage regeneration *in vitro*.

In addition to the type of ECM ligand coating, the ligand retention level, representing ligand density on the PGD surface, is another key parameter that influenced chondrocyte function during monolayer culture. Studies have shown that the high concentration of collagen type I and hyaluronic acid substrate increased cartilaginous matrix deposition compared with a low ligand density [19,55]. However, although saturated hyaluronic acid on 2D PGD presented a beneficial effect on chondrocyte redifferentiation (Figure 3), this effect could be limited by the reduced ligand retention during the culture. To avoid the potential beneficial effect of a high seeding density on chondrocyte function, we used a low-cell-density model for the VD coating groups, and the ligand retention was accurately controlled by our methodology (Figure 4a). Unlike collagen, we found that hyaluronic acid had a very low retention level after 24 h immersion in PBS because the hydrophilic character, excellent water solubility, rapid degradation, and non-crosslinked nature of hyaluronic acid usually led to poor localization and retention during cell culture [56,57,58]. Interestingly, incorporating hyaluronic acid into collagen improved its retention on PGD but also suppressed hAC’s anabolic activity (Figure 4d), suggesting that there were synergetic influences between ligand composition and ligand retention on chondrocyte’s ECM production. Coating with either HyA or Col I alone induced a dose-dependent response (positive correlation) in hAC’s anabolic activity to the retention of the ligand on PGD. This finding was also supported by other studies. For example, a high concentration of hyaluronic acid coating was found to be able to reduce the acidity of degradation products of polyester scaffold, and further attenuate an acute inflammatory reaction *in vivo* and improve the biocompatibility of the polyester [16]. However, the combination of Col I and HyA, even with a higher HyA retention level, was not conducive to a higher ECM accumulation by hACs compared with HyA alone. A previous study has shown that coating hyaluronic acid onto polymer with the same retention level but in a different way changes the other scaffold parameters, e.g., surface morphology, which also exhibit an impact on articular chondrocyte behavior [59]. We found that ligand composition determined the phenotypic characters of chondrocyte, such as cell morphology (Figure 4b) and co-localization of the crucial macromolecules (Figure 4e), and, more importantly, this effect was augmented by ligand density due to the increased number of cells that presented corresponding characteristics on PGD with a higher ligand density. Moreover, histological results revealed that the ligand density could influence the dimension and thickness of 2D aggrecan deposition during monolayer culture, thus altering the amount of ECM produced by hACs on PGD (Figure 4f). In the meantime, ligand composition also participated in regulating the distribution of the neo-matrix (Figure 4f), probably because it implicates the transduction of mechanical signals in chondrocytes and causes changes in intracellular pathways and cell–cell interaction [4,11]. Therefore, these results suggest that there are combinatorial effects of ligand composition and density on human articular chondrocyte function, which means the effect of these ligands on cell anabolic activity is more complex than previously thought and requires further study.

## 5. Conclusions

In this study, we endeavored to develop a simplified but effective ligand coating strategy for PGD and explore the effects of coating parameters on chondrocyte behavior. We found that there was a combinatorial effect of ligand coating density and composition on chondrocyte shape and ECM production. The morphology of hACs on PGD in short-term culture is mainly regulated by the composition of ligand coating, and this effect is augmented by ligand density. The amount and distribution of produced ECM in long-term culture is regulated by not only the type or combination of ligand coating, but also the ligand retention level on PGD. The ligand coating on PGD is a viable strategy to increase the substrate’s hydrophilicity and biocompatibility, improve chondrocyte function, and promote chondrocyte redifferentiaion, and, more importantly, these could be tailored by ligand composition and density. Overall, the synergetic effect of ligand parameters suggests a potential guideline for optimizing surface micro-environmental cues on PGD in both 2D and 3D culture and investigating the mechanistic regulation of surface parameters on chondrocyte function for tissue engineering purposes. This will be a significant push towards the clinical application of chondrocytes-based repair for cartilage defects using PGD or other elastomers with similar polyester properties.

## Figures and Tables

**Table 1:** Mass of ligand coating with different coating compositions.

Ligand Labels	Mass on PGD (µg)
C, Col I: Collagen type I	50, 100, 200, or 400
H, HyA: Hyaluronic acid	62.5, 125, 250, or 500
C+H: First layer of C, then second layer of H	C: 200; H: 250 or 500

## Data Availability

The data presented in this study are available on request from the corresponding author.
